# The defense response in *Arabidopsis thaliana* against *Fusarium sporotrichioides*

**DOI:** 10.1186/1477-5956-10-61

**Published:** 2012-10-30

**Authors:** Tomoya Asano, Makoto Kimura, Takumi Nishiuchi

**Affiliations:** 1 Division of Functional Genomics, Advanced Science Research Center, Kanazawa University, 13-1 Takaramachi, Kanazawa, 920-0934, Japan; 2 Equipment Support Promotion office, Advanced Science Research Center, Kanazawa University, Kanazawa, 920-1192, Japan; 3 Plant & Microbial Metabolic Engineering Research Unit, Discovery Research Institute (DRI), RIKEN, 2–1 Hirosawa, Wako, Saitama, 351-0198, Japan; 4 Division of Life Science, Graduate School of Natural Science and Technology, Kanazawa University, Kanazawa, 920-1192, Japan

**Keywords:** *Arabidopsis thaliana*, Defense response, *Fusarium sporotrichioides*, T-2 toxin, MAP kinase, GSTs, Superoxide dismutase, Ascorbate peroxidase

## Abstract

**Background:**

Certain graminaceous plants such as *Zea mays* and *Triticum aestivum* serve as hosts for *Fusarium sporotrichioides*; however, molecular interactions between the host plants and *F. sporotrichioides* remain unknown. It is also not known whether any interaction between *Arabidopsis thaliana* and *F. sporotrichioides* can occur. To understand these interactions, we performed proteomic analysis.

**Results:**

Arabidopsis leaves and flowers were inoculated with *F. sporotrichioides*. Accumulation of *PLANT DEFENSIN1.2* (*PDF1.2*) and *PATHOGENESIS RELATED1* (*PR1*) mRNA in Arabidopsis were increased by inoculation of *F. sporotrichioides.* Furthermore, mitogen-activated protein kinase 3 (MPK3) and mitogen-activated protein kinase 6 (MPK6), which represent MAP kinases in Arabidopsis, were activated by inoculation of *F. sporotrichioides.* Proteomic analysis revealed that some defense-related proteins were upregulated, while the expression of photosynthesis- and metabolism-related proteins was down regulated, by inoculation with *F. sporotrichioides.* We carried out the proteomic analysis about upregulated proteins by inoculation with *Fusarium graminearum.* The glutathione *S*-transferases (GSTs), such as GSTF4 and GSTF7 were upregulated, by inoculation with *F. graminearum*-infected Arabidopsis leaves. On the other hand, GSTF3 and GSTF9 were uniquely upregulated, by inoculation with *F. sporotrichioides.*

**Conclusions:**

These results indicate that Arabidopsis is a host plant for *F. sporotrichioides*. We revealed that defense response of Arabidopsis is initiated by infection with *F. sporotrichioides*.

## Background

Fusarium head blight (FHB) is a severe disease that affects cereal crops worldwide [1,2]. This disease is caused by *Fusarium* species, such as *Fusarium sporotrichioides*, *F. graminearum*, and *F. culmorum*. The soil-borne pathogen *F. sporotrichioides* is often observed in cold climates, such as in northern Japan, the northern USA, northern Europe, and Russia [3]. *F. sporotrichioides* was first isolated from corn in France and was identified as *F. tricinctum* NRRL 3229 [3]; it was later determined that this strain included both *F. sporotrichioides* and *F. tricinctum*. Similarly, the *F. sporotrichioides* IFO 9955 strain was identified from a bean hull by Ueno et al. [3], although it had previously been misidentified as *F. solani*[3]*.* It has been reported that overwintered cereals colonized by *F. sporotrichioides* caused the deaths of approximately 1,100 people in the erstwhile USSR during World War II [3].

Some *Fusarium* species also produce trichothecene mycotoxins, which are known to inhibit protein synthesis in eukaryotes [4,5]. Trichothecenes encompass many molecular species, which can be classified into 4 major groups (types A–D). Among these species, the type A (i.e., T-2 toxin) and type B (i.e., deoxynivalenol [DON]) trichothecenes are distinguished by the absence or presence of a ketone group at the C-8 position, respectively [6]. Trichothecenes are often found in cereal grains and cereal-derived commodities [6]. T-2 toxin has been reported to be approximately 10 times more toxic to mammals and plants than the type B trichothecenes, such as DON [7]. The *tri5* mutant of *F. graminearum*, which cannot produce DON, can infect wheat florets and spikes, but it has decreased virulence against host plants [8]. Bai et al. suggested that DON supports the infection of *F. graminearum* in wheat [8]*.* On the other hand, the expression of *PLANT DEFENSIN1.2* (*PDF1.2)* and *PATHOGENESIS RELATED1 (PR1)* mRNAs in Arabidopsis were induced by T-2 toxin [9]. Mitogen-activated protein kinase 3 (MPK3) and mitogen-activated protein kinase 6 (MPK6) in Arabidopsis have also been shown to be activated by T-2 toxin [9]. Nishiuchi et al. suggested that T-2 toxin possesses an elicitor-like activity [9].

The elicitors from *Fusarium* species induce the defense response of *Arabidopsis* cell suspension cultures [10,11]. The chitosan from *F. moniliforme* induced the expression of glutathione *S*-transferases (GSTs) in Arabidopsis, which is marker protein of pathogen defense response [11]. Moreover, some *Fusarium* species produce the mycotoxins, such as fumonisins [12]. The fumonisins are produced by *F. moniliforme*, [12]*F. proliferatum, F. anthophilum, F. dlamini, F. napiforme* and *Alternaria alternata* f. sp. *lycopersici*[12]. The fumonisins acts as an inhibitor of sphingosine *N*-acetyltransfearase [12], and the fumonisin B1 (FB1) causes the program cell death (PCD) in plants and animals [13]. Furthermore, the expression of *PR* gene*s* and *PDF1.2* were induced by FB1 [13].

The upregulation of GSTs has been reported in type B-producing *F. graminearum*-infected wheat flowers [14]. GSTs function in the detoxification of both endogenous and xenobiotic compounds [15]. Gardiner et al. found evidence for nonenzymatic formation of DON-GSH conjugates *in vitro* using both liquid chromatography-mass spectrometry and nuclear magnetic resonance analysis [16] and suggested that GST is involved in DON detoxification [16]. Furthermore, disease symptoms manifested by Arabidopsis in response to *F. graminearum* are related to ethylene signaling [17]; other interactions between host plants and some *Fusarium* species have been reported. However, it is unknown whether there is an interaction between *A. thaliana* and *F. sporotrichioides*.

Proteomics is a tool to gain information on the protein levels, and it has frequently been used to study plant diseases [18,19]. Mukherjee et al. suggested that proteomic analysis of the defense response to *Alternaria brassicicola* can be compared to other types of plant–pathogen interactions and leaf senescence in Arabidopsis [20]. The interaction of *F. graminearum* with some host plants has also previously been investigated by proteomic analysis. For instance, *Triticum aestivum* interacts with *F. graminearum*[21]. In this regard, Zhou et al. suggested that proteins related to jasmonic acid signaling pathways, PR protein, amino acid synthesis, and nitrogen metabolism were upregulated by inoculation of the plant with *F. graminearum*[21]*.* The defense response of *Hordeum vulgare* is also elicited by infection with *F. graminearum*[22]. Geddes et al. suggested that FHB caused increases in proteins associated with the oxidative burst and oxidative stress response, such as malate dehydrogenase, peroxidases, and PR protein [22]. However, although *F. sporotrichioides* produces the strong T-2 toxin, the molecular interaction between host plants and *F. sporotrichioides* is not understood.

In this paper, we report that Arabidopsis can act as a host to *F. sporotrichioides.* We demonstrate the defense response in Arabidopsis leaves caused by inoculation with *F. sporotrichioides*; furthermore, a proteomic analysis revealed induction of some defense response proteins by inoculation with *F. sporotrichioides*.

## Results and discussion

**Figure 1:**
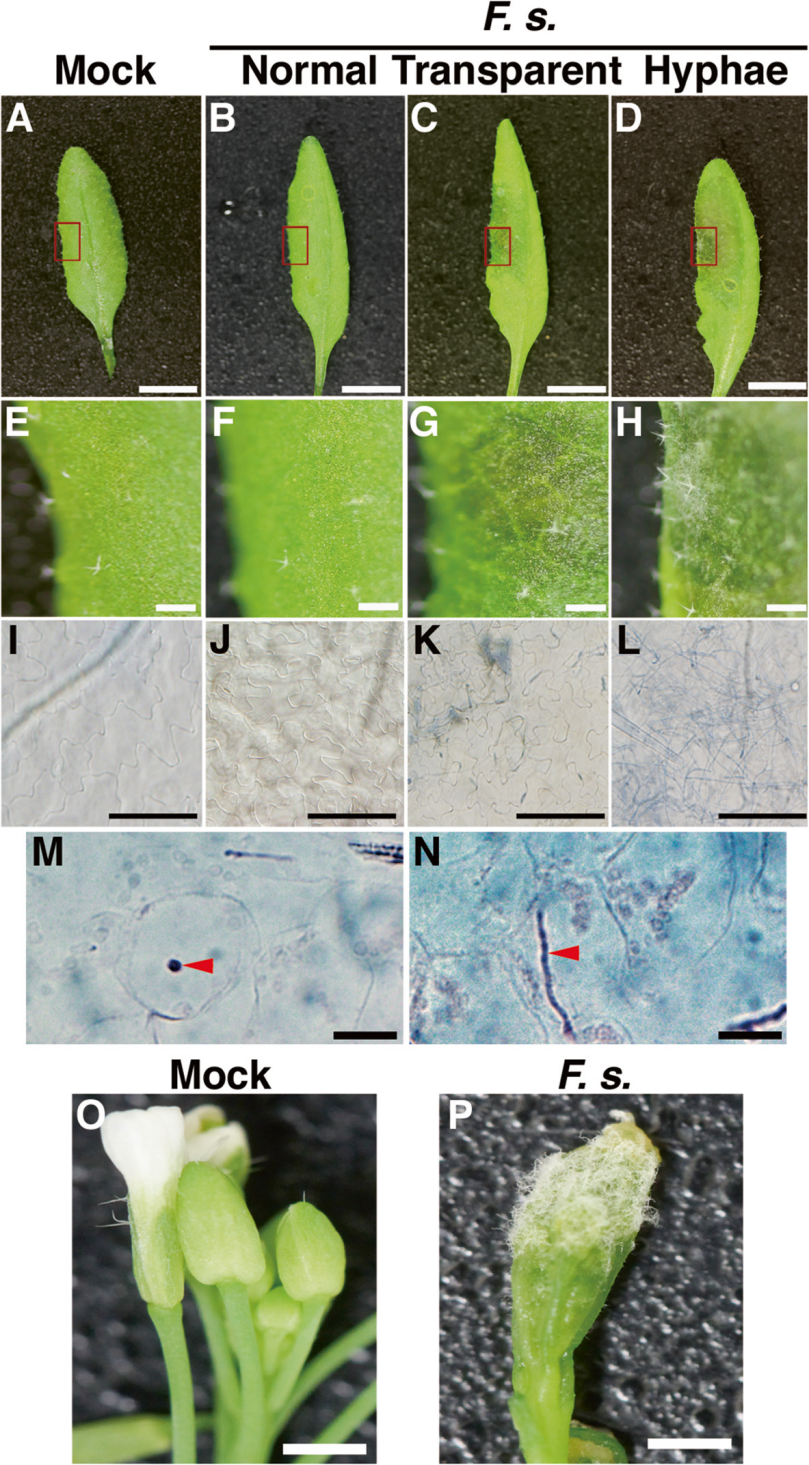
**Inoculation of Arabidopsis leaves and flower buds with *Fusarium sporotrichioides.*** Photographs of mock-treated (**A**, **E**, **I**) and conidial suspension–treated Arabidopsis leaves (**B**–**D**, **F**–**H**, **J**–**L**) at 2 dpi. (**E**–**H**) represents magnification of the inner box of panels **A**–**D**, respectively. Trypan blue staining of mock-treated leaves (**I**) and leaves inoculated with *F. sporotrichioides* (**J**–**L**). (**M**, **N**) Cross-sections of Trypan blue staining of leaves inoculated with *F. sporotrichioides*. Arrowheads show the hyphae. Photographs of mock-treated (**O**) and conidial suspension–treated flower buds (**P**) at 2 dpi. The scale bars indicate 1 cm (**A**–**D**), 1 mm (**E**–**H**, **O**, **P**), and 100 μm (**I**–**N**).

In this study, we first examined the virulence of *F. sporotrichioides* in Arabidopsis rosette leaves and flower buds. Makandar et al. revealed that efficient infection and disease by *F. graminearum* occurs when the fungus is infiltrated into *Arabidopsis* leaves [23]. *F. graminearum* H3 and *F. graminearum* ZEA-1 strains [24] were reported to show the pathogenicity to cereals in a farm (unpublished data). *F. graminearum* H3 produces DON, and *F. graminearum* ZEA-1 produces zearalenone (ZEA) and DON [24]. We infiltrated with highly concentrated conidial suspensions (1 × 10^5^ conidia/mL) of plant-fungal *F. graminearum* H3 or ZEA-1 into Arabidopsis leaves. The both infectious hyphae of *F. graminearum* ZEA-1 and *F. graminearum* H3 were observed at a rate of about 50% in inoculated Arabidopsis leaves at 2 days post inoculation (dpi). These results indicate that *F. graminearum* ZEA-1 and *F. graminearum* H3 are phytopathogens to Arabidopsis. Next, we inoculated the Arabidopsis leaves with highly concentrated conidial suspensions (1 × 10^6^ conidia/mL) of *F. sporotrichioides* (Figure 1). At 2 dpi, normal leaves were observed in about 72% of the Arabidopsis leaves that had been infiltrated with *F. sporotrichioides* (Figures 1B, F) compared with mock treatment leaves (Figures 1A, E). However, transparent regions were observed in about 21% of those leaves (Figures 1C, G), and infectious hyphae were observed in about 7% of those leaves (Figures 1D, H). On the other hand, hyphae of *F. sporotrichioides* were also observed in all the inoculated Arabidopsis flower buds (Figures 1O, P). Trypan blue staining showed that infectious hyphae were present in the leaves infiltrated with *F. sporotrichioides* at 2 dpi (Figures 1I-N). Thus, these results indicate that Arabidopsis is a host plant of *F. sporotrichioides.*

We next examined whether T-2 toxin accumulated in *F. sporotrichioides*–inoculated leaves. The type B trichothecenes, such as DON, were detected by matrix-assisted laser desorption/ionization time-of-flight mass spectrometry (MALDI-TOF MS) [25]. Thus, we then established a method for quantification of T-2 toxin using MALDI-TOF MS. We tested sodium azide as a matrix for detecting T-2 toxin. Additional file 1 shows that T-2 toxin was successfully ionized by sodium azide at 489.2 Da, corresponding to the sodium adduct of the toxin. In addition, the detection limit for T-2 toxin was approximately 50 fmol (data not shown). Another type A trichothecene, diacetoxyscirpenol (DAS), was also detected by the same method ( Additional file 1). Because DAS was not detected in any of the samples inoculated with *F. sporotrichioides*, we used DAS as an internal control to quantify T-2 toxin in *F. sporotrichioides*–inoculated tissues. We then measured the concentration of T-2 toxin in rosette leaves inoculated with conidial suspensions of *F. sporotrichioides* by the infiltration method. T-2 toxin appeared to have accumulated in the *F. sporotrichioides*–inoculated leaves to an average level of 17.6 ± 6.84 ng per leaf (n = 6), whereas T-2 toxin was not detected in the mock-treated leaves. The accumulation of this volume of T-2 toxin in Arabidopsis leaves caused the cell death and defense response [9]. Thus, the accumulation of T-2 toxin in the *F. sporotrichioides*–inoculated leaves was sufficient to contribute to their virulence.

**Figure 2:**
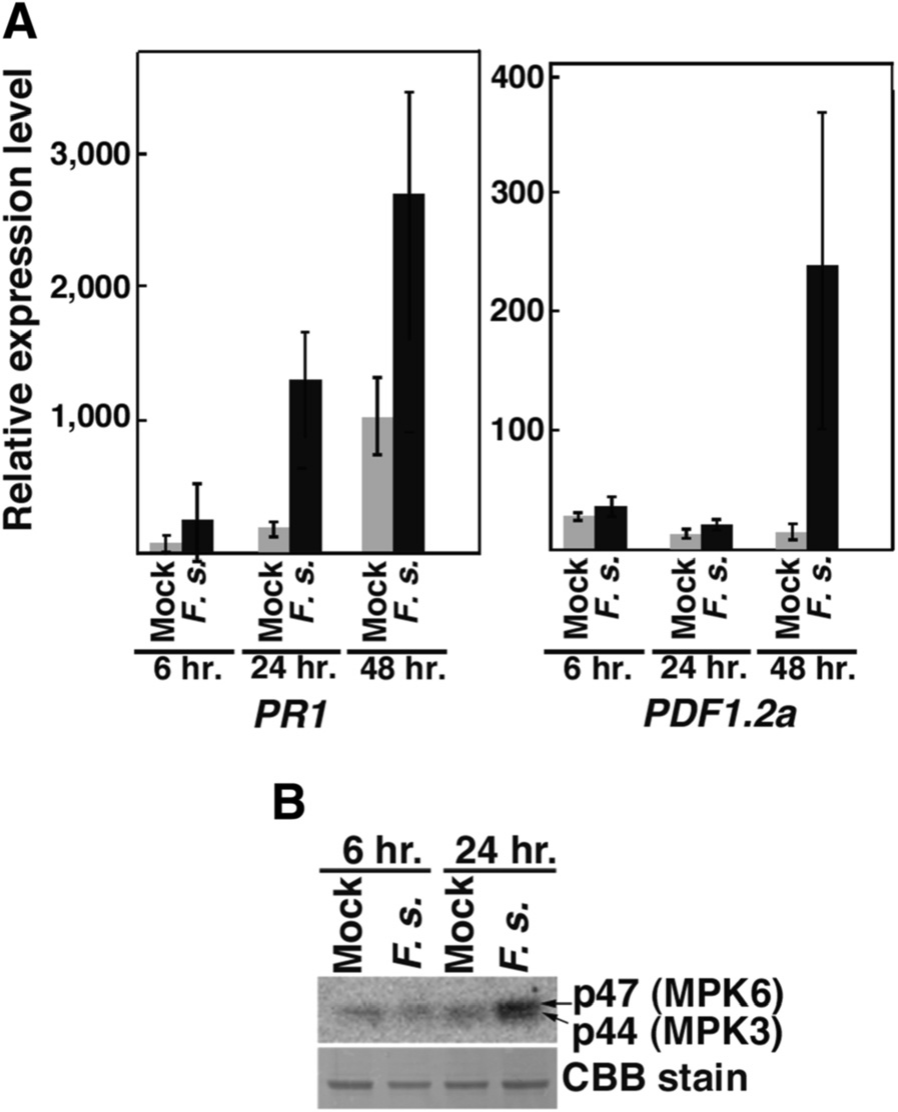
**The expression pattern of *PDF1.2a *and *PR1 *mRNA in Arabidopsis by inoculation of *F. sporotrichioides.*** (**A**) The expression analysis of *PR1* and *PDF1.2a* mRNA in Arabidopsis after inoculation. *ACT2* and *ACT8* were used as reference genes. These experiments were repeated 3 times. (**B**) Activity of MPK3 and MPK6 in response to inoculation with *F. sporotrichioides*. Upper panel: results of an in-gel kinase assay. Lower panel: CBB staining of the loading control. The amplification efficiency for qRT-PCR of *PR-1, PDF1.2* and *ACT2, 8* were 91.6, 86.7 and 98.2%, respectively. These experiments were repeated 3 times.

After infiltration inoculation with highly concentrated microconidial suspensions, infectious *F. sporotrichioides* hyphae were observed in Arabidopsis leaf cells (Figures 1M, N; arrowheads). Arabidopsis has previously been demonstrated to show interaction with *F. sporotrichioides* in that the expression of *PR1* and *PDF1.2a* mRNAs in Arabidopsis were induced by inoculation with fumonisins-producing *F. moniliforme*[13], *F. oxysporum*[26], *F. graminearum*[9]. We therefore investigated the amount of *PR1* and *PDF1.2a* mRNAs of *F. sporotrichioides*-inoculated Arabidopsis leaves by quantitative real-time RT-PCR (qRT-PCR) analysis. The *PR1* mRNAs were induced by inoculation with *F. sporotrichioides* at 24 and 48 h post inoculation (Figure 2A)*.* The *PDF1.2a* were induced by inoculation with *F. sporotrichioides* at 48 h post inoculation (Figure 2A)*.*

Moreover, the activities of p47 (MPK6) and p44 (MPK3) have been shown to be activated by bacterial and fungal pathogen–associated molecular patterns (PAMPs) during plant–pathogen interactions [27]. MPK6 and MPK3 have also been shown to be activated by T-2 toxin and DON [9]. To investigate the defense response of Arabidopsis to *F. sporotrichioides*, we performed an in-gel kinase assay using myelin basic protein. The activities of MPK6 and MPK3 were not different between *F. sporotrichioides*–inoculated and mock-treated leaves by 6 h post inoculation (Figure 2B). However, the activities of MPK6 was increased by inoculation of *F. sporotrichioides* compared to mock treatment by 24 h post inoculation (Figure 2B)*.* On the other hand, the activation of MPK3 was weak compared with MPK6 (Figure 2B). These results indicate that the defense response of Arabidopsis is induced by *F. sporotrichioides* infection*.*

**Figure 3:**
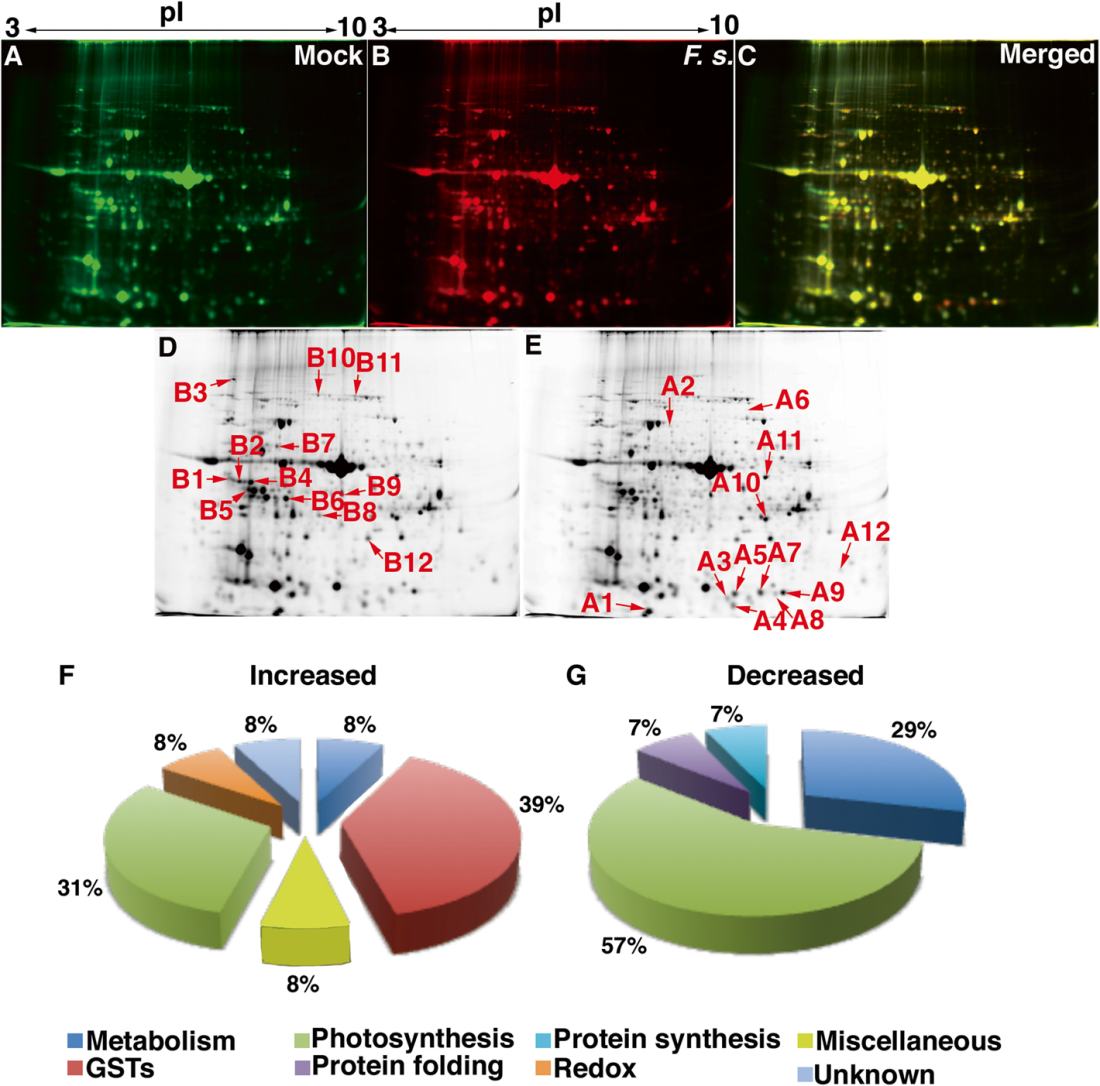
**Proteomic analysis of Arabidopsis leaves inoculated with a conidial suspension of *F. sporotrichioides*.** (**A**) Protein spots of mock-treated samples (Cy3-labeled total protein). (**B**) Protein spots of *F. sporotrichioides*–inoculated leaves (Cy5-labeled total protein). (**C**) Merge of (**A**) and (**B**) images. Spots of downregulated (**D**) and upregulated (**E**) proteins in *F. sporotrichioides*–inoculated leaves are shown. The spot numbers correspond to the results of protein identification (Table 1). This experiment was repeated 3 times. Functional classification of the identified proteins is shown. (**F**) The proteins increased and (**G**) decreased in response to inoculation with *F. sporotrichioides*.

To profile the defense response of Arabidopsis leaves against *F. sporotrichioides*, we performed a proteomic analysis in mature Arabidopsis leaves inoculated with *F. sporotrichioides* by the infiltration method. For this purpose, we performed two-dimensional (2D) difference gel electrophoresis. Total protein was extracted from mock-treated and *F. sporotrichioides*–inoculated Arabidopsis leaves, and fluorescently labeled with Cy3 and Cy5, respectively. The resulting fluorescently labeled proteins were mixed and subjected to 2D electrophoresis on the same gel. As shown in Figures 3A–E, 24 protein spots exhibited significantly different expression patterns (upregulated or down regulated) in Arabidopsis leaves inoculated with *F. sporotrichioides*. These protein spots were digested with trypsin, and the resulting peptides were identified by MALDI-TOF/TOF analyzer. Many proteins belonging to GSTs were increased in *F. sporotrichioides*–inoculated Arabidopsis leaves (Figure 3F). Table 1 shows that the levels of 5 GSTs from spots A3, A5, A7, A8, and A9 were upregulated to 2.66, 3.28, 1.82, 204, and 1.35-fold by inoculation, respectively (Table 1). The spots A5 (GSTF4a) and A7 (GSTF4b) were identified as same GSTF4. Some post-translational modifications of GSTs have been identified in Arabidopsis [28]. This different position of GSTF4 might be the post-translational modification. GSTs in Arabidopsis were used as marker protein of pathogen defense response [11]. GSTs are known to function in the maintenance of the redox state and the detoxification of toxins in many species [29]. The formation of DON-glutathione (DON-GSH) conjugates is likely to play a role in the detoxification of DON [16]. Therefore, these enhanced GSTs might be involved in the detoxification of T-2 toxin.

**Table 1:** **Identification of protein spots that were differentially expressed after the inoculation of Arabidopsis leaves with *F. sporotrichioides***

**Spot No.**	**Cov. (%)**^***a***^	**Total**^***b***^	**Description**	**Species**	**Protein ID**^***c***^	**Fusarium/Mock**^***d***^
A1	38.2	14.0	LHCA1	*A. thaliana*	gi|15233115	6.15
A1	35.2	10.4	Putative H^+^-transporting ATP synthase	*A. thaliana*	gi|18491181	6.15
A2	8.10	1.06	Transketolase-like protein	*A. thaliana*	gi|7329685	62.4
A3	30.8	6.00	Glutathione S-transferase 6 (GSTF3)	*A. thaliana*	gi|15218640	2.66
A4	29.5	12.0	Superoxide dismutase	*A. thaliana*	gi|4455253	1.34
A5	59.9	18.8	Glutathione S-transferase (GSTF4a)	*A. thaliana*	sp|P46422|GSTF4_ARATH	3.28
A6	9.00	2.06	Alpha-xylosidase	*A. thaliana*	sp|Q9S7Y7|XYL1_ARATH	22.6
A7	57.5	15.9	Glutathione S-transferase (GSTF4b)	*A. thaliana*	sp|P46422|GSTF4_ARATH	1.82
A8	42.1	12.5	Glutathione S-transferase 11 (GSTF9)	*A. thaliana*	sp|Q9SRY5|GSTF9_ARATH	204
A9	54.4	24.0	Glutathione S-transferase 9 (GSTF7)	*A. thaliana*	gi|15224581	1.35
A10	34.1	11.5	ATP synthase gamma chain	*A. thaliana*	gi|5708095	1.15
A11	34.6	15.7	Formate dehydrogenase	*A. thaliana*	gi|15241492	1.38
A12	12.5	3.20	putative ascorbate Peroxidase APX4	*A. thaliana*	gi|31980500	1.95
B1	44.4	18.4	Rubisco activase	*A. thaliana*	gi|18405145	0.65
B2	44.3	24.4	Rubisco activase	*A. thaliana*	gi|18405145	0.44
B3	10.4	11.9	Embryo defective 2726	*A. thaliana*	gi|18417320	0.57
B4	43.0	22.2	Rubisco activase	*A. thaliana*	gi|18405145	0.76
B5	26.7	10.0	Glutamine synthetase 2	*A. thaliana*	gi|15238559	0.67
B6	38.5	20.0	Rubisco activase	*A. thaliana*	gi|30687999	0.70
B7	17.1	13.1	Chaperonin, putative	*A. thaliana*	gi|15231255	0.88
B7	18.3	6.01	ATP synthase CF1 alpha subunit	*A. thaliana*	gi|7525018	0.88
B8	19.5	6.15	ATP synthase gamma chain, chloroplast precursor	*A. thaliana*	gi|5708095	0.54
B8	7.80	4.00	Indole-3-acetonitrile nitrilase	*A. thaliana*	gi|30692067	0.54
B9	12.9	6.09	Glyceraldehyde-3-phosphate dehydrogenase B subunit	*A. thaliana*	gi|336390	0.96
B10	21.7	26.3	*Arabidopsis thaliana* glycine Decarboxylase P-protein 2	*A. thaliana*	gi|15225249	0.78
B11	24.9	24.1	*Arabidopsis thaliana* glycine Decarboxylase P-protein 1	*A. thaliana*	gi|15234036	0.65
B12	28.7	10.7	Ferredoxin-NADPH(+)-Oxidoreductase 2	*A. thaliana*	gi|15223753	0.89

^***a***^Cov. (%); Coverage: the percent ratio of all amino acids from valid peptide matches to the total number of amino acids in the protein. ^***b***^Total ProtScore was calculated from confidence for all peptides detected for a given identified protein. ^***c***^Protein ID indicates the accession number of the protein. ^***d***^The ratio of *Fusarium*/Mock shows the fold-change in expression levels between *Fusarium*-inoculated leaves and mock-treated leaves for each protein. Data are representative of 3 independent experiments.

Moreover, superoxide dismutase (SOD) from spot A4 was upregulated to 1.3-fold by inoculation (Table 1). The ascorbate peroxidase (APX) protein from spot A12 was enhanced to 2.0-fold by inoculation (Table 1). It is known that T-2 toxin causes the accumulation of hydrogen peroxide in Arabidopsis leaves [9]. Accumulated reactive oxygen species (ROS) are detoxified by SOD and APX [30]. Thus, the induction of SOD and APX by inoculation of *F. sporotrichioides* might also be associated with the detoxification of superoxide in Arabidopsis leaves [29].

**Figure 4:**
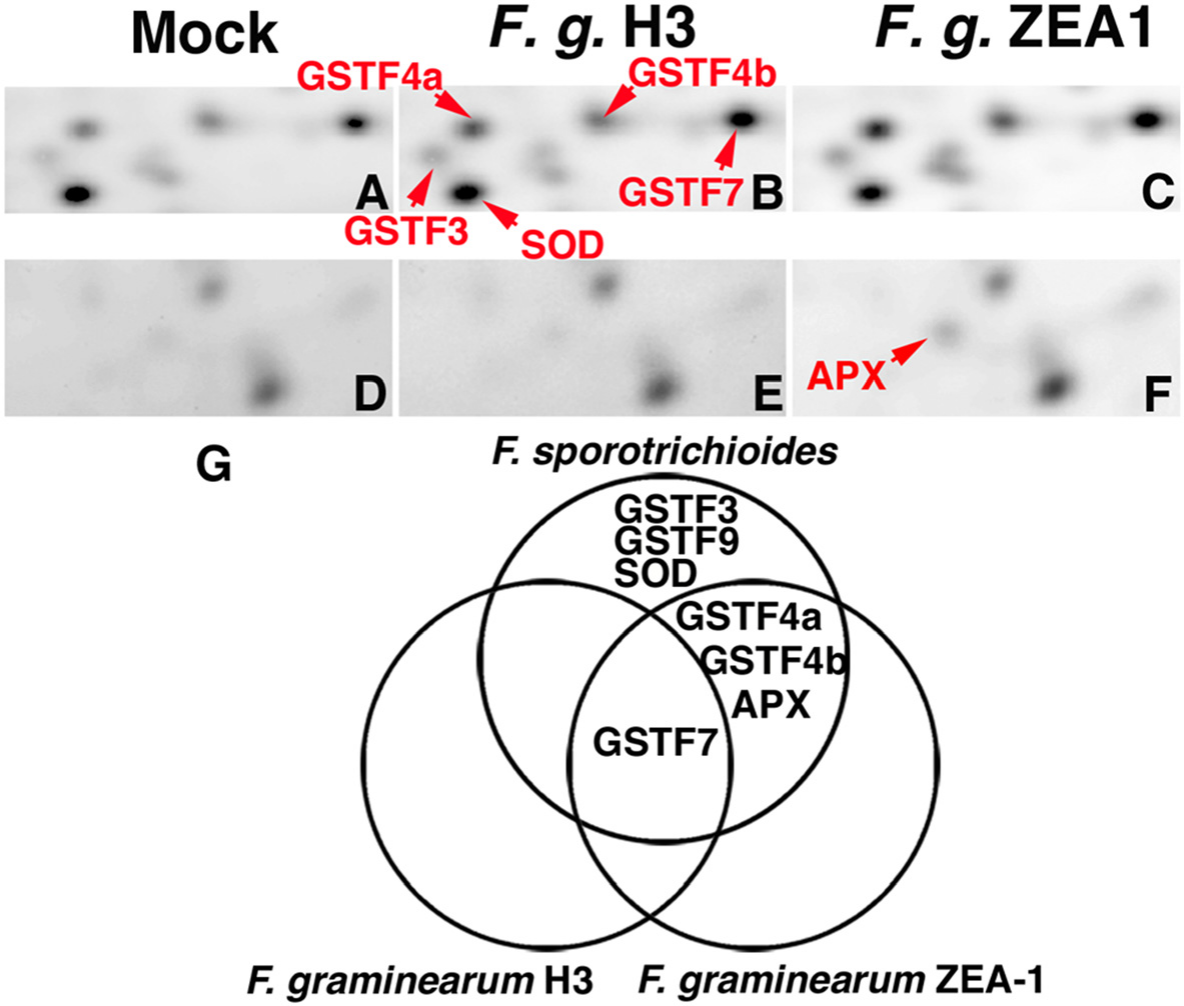
**The expression pattern of GSTs, APX and SOD in Arabidopsis leaves inoculated with a conidial suspension of *F. graminearum *H3 and ZEA-1.** (**A**, **D**) Protein spots of mock-treated samples (Cy2-labeled total protein). (**B**, **E**) Protein spots of *F. graminearum* H3-inoculated leaves (Cy3-labeled total protein). (**C**, **F**) Protein spots of *F. graminearum* ZEA-1-inoculated leaves (Cy5-labeled total protein). This experiment was repeated 3 times. (**G**) Venn diagram showing of expression pattern of GSTs, APX and SOD proteins in Arabidopsis leaves inoculated with a conidial suspension of *F. sporotrichioides*, *F. graminearum* H3 and ZEA-1.

To compare the defense response in Arabidopsis to *F. sporotrichioides* and other *Fusarium* species*,* we carried out the proteomic analysis with inoculation of two known plant-fungal *F. graminearum. F. graminearum* H3 and ZEA-1 were used in this study. Total proteins of mock, *F. graminearum* H3 and *F. graminearum* ZEA-1-treated Arabidopsis leaves were fluorescently labeled with Cy2, Cy3 and Cy5, respectively. The expression of GSTF7 was upregulated to 1.20-fold by inoculation of *F. graminearum* H3 compared with mock treatment (Figure 4A, B, Table 2). GSTF4a, GSTF4b, GSTF3, GSTF9, APX and SOD were not increased with inoculation of *F. graminearum* H3 (Figure 4A, B, D, E, Table 2). On the other hand, GSTF4a, GSTF4b, GSTF7 and APX were upregulated to 1.48, 1.19, 1.36 and 2.17-fold by inoculation of *F. graminearum* ZEA-1, respectively (Figure 4A, C, D, F, Table 2). The extracellular matrix from *F. moniliforme* contains various elicitors to Arabidopsis, such as chitosan [11]. The expression of GSTF7 was enhanced by inoculation of *F. sporotrichioides*, *F. graminearum* H3 and *F. graminearum* ZEA-1 (Figure 4G). These results indicate that GSTF7 might be enhanced by common elicitors of *Fusarium* species. On the other hand, the expression of GSTF4a, GSTF4b and APX were increased with *F. sporotrichioides* and *F. graminearum* ZEA-1 (Figure 4G). We suggest that GSTF4a, GSTF4b, GSTF7 and APX were enhanced by common elicitors of *F. sporotrichioides* and *F. graminearum* H3.

**Table 2:** **The protein spots that were differentially expressed after the inoculation of Arabidopsis leaves with two *F. graminearum *H3 and ZEA-1 strains**

**Spot No.**	**Description**	**Protein ID**^***a***^	***F. graminearum *H3/Mock**^***b***^	***F. graminearum *ZEA-1/Mock**^***b***^
A3	Glutathione S-transferase 6 (GSTF3)	gi|15218640	1.03	1.13
A4	Superoxide Dismutase	gi|4455253	1.10	1.10
A5	Glutathione S-transferase (GSTF4a)	sp|P46422|GSTF4_ARATH	1.13	1.48
A7	Glutathione S-transferase (GSTF4b)	sp|P46422|GSTF4_ARATH	1.03	1.19
A8	Glutathione S-transferase 11 (GSTF9)	sp|Q9SRY5|GSTF9_ARATH	N. D.	N. D.
A9	Glutathione S-transferase 9 (GSTF7)	gi|15224581	1.20	1.36
A12	Putative Ascorbate Peroxidase APX4	gi|31980500	0.80	2.17

^***a***^Protein ID indicates the accession number of the protein. ^***b***^The ratio of *Fusarium*/Mock shows the fold-change in expression levels between *Fusarium*-inoculated leaves and mock-treated leaves for each protein. N. D. shows the not detected the spot. Data are representative of 3 independent experiments.

Also, the expression of GSTF3, GSTF9 and SOD were uniquely enhanced by inoculation with *F. sporotrichioides* (Figure 4G). *F. sporotrichioides* produces trichothecenes, which is T-2 toxin [6]. T-2 toxin from *F. sporotrichioides* induces the defense response in Arabidopsis [9]. The expression of GSTF3, GSTF9 and SOD might be enhanced by T-2 toxin. We suggest that the defense response in Arabidopsis against *F. sporotrichioides* causes by the various elicitors.

Conversely, the expression of photosynthesis- and metabolism-related proteins such as Rubisco activase, ATP synthase, and ferredoxin-NADP^+^ oxidoreductase was downregulated in Arabidopsis leaves by inoculation with *F. sporotrichioides* (Figure 3G). Rubsico activase from spots B1, B2, B4, and B6 were downregulated 0.65, 0.44, 0.76, and 0.70-fold by inoculation (Table 1). Rubisco activase is required to allow the regeneration of the critical carbamate in the active site of Rubisco [31]. Moreover, glutamine synthetase 2 was decreased to 0.67-fold by inoculation (Table 1). Glutamine synthetase is one of the essential enzymes for glutamine production [32]. We propose that the activation of the defense-signaling pathway was enhanced, while the expression of photosynthesis- and metabolism-related proteins was suppressed by inoculation with *F. sporotrichioides*.

Five days after inoculation with *F. graminearum*, GSTs and SOD were upregulated or induced, and Rubisco activase were downregulated [14,21]. These results indicate that the interaction between Arabidopsis and *F. sporotrichioides* is similar to the interaction between wheat and *F. graminearum* at the proteomic level*.*

## Conclusions

Some phytopathogenic *Fusarium* species, including *F. sporotrichioides*, are known to produce type A trichothecenes, such as T-2 toxin. However, the interactions between type A trichothecene–producing *Fusarium* species and plants have not been well studied. In this study, virulence of *F. sporotrichioides* was observed in Arabidopsis leaves after inoculation. Even when the Arabidopsis leaves were infiltrated with conidial suspensions of *F. sporotrichioides*, invasive hyphae were observed in the leaves. *PR1* and *PDF1.2* mRNA were induced by inoculation of *F. sporotrichioides.* MPK3 and MPK6, which are MAP kinases in Arabidopsis, were also activated in 24 h by *F. sporotrichioides.* Proteomic analysis revealed that some defense-related proteins, including 5 GSTs, SOD, and APX, were increased in the *F. sporotrichioides*–infiltrated leaves*.* Also, GSTF3, GSTF9 and SOD were uniquely enhanced by inoculation of *F. sporotrichioides*. These results indicate that a defense response is caused in Arabidopsis leaves by infection with *F. sporotrichioides.*

## Methods

### Plant and fungal growth

The Columbia (Col-0) ecotype of *A. thaliana* (L.) Heynh. was used in this study. Arabidopsis seeds were sown in soil, placed at 4°C in the dark for 2 days, and subsequently grown at 22°C under a 16/8-h light/dark cycle. *F. sporotrichioides* strain IFO 9955 (previously misidentified as *Fusarium solani*) was used in this study [33]. *F. graminearum* H3 (MAFF101551) and ZEA-1 strains were used in this study. The fungi was grown at 22°C under weak light on Synthetic Low Nutrient (SN) agar medium. The production of microconidia was induced by SN liquid medium (0.1% KH_2_PO_4_, 0.1% KNO_3,_ 0.1% MgSO_4_·7H_2_O, 0.05% KCl, 0.02% glucose, 0.02% sucrose) [34].

### Fungal inoculation

For the preparation of conidia, *F. sporotrichioides* was cultured in SN liquid medium with shaking for 2 days at 22°C, in constant darkness. The conidia were collected by centrifugation (14,000 *g* at room temperature for 5 min) and were washed with phosphate-buffered saline (PBS) at least 3 times. The collected conidia were suspended in PBS and the number of conidia counted using a hemocytometer. For the infiltration inoculation, a conidial suspension (1 × 10^6^ conidia/mL) or PBS (mock) without detergent was injected into the abaxial sides of the leaves with a needleless syringe [35]. The inoculated plants were incubated in a chamber under about 100% relative humidity, at 22°C, and a 16/8-h light/dark cycle.

### Trypan blue staining

The hyphae of inoculated leaves were stained by previously described solution [36]. After staining, leaves were washed in chloral hydrate [36].

### The quantitative real-time RT-PCR

Total RNA was isolated using the Plant RNA Isolation Mini Kit (Agilent Technologies, CA, USA). First-strand cDNA was synthesized using the PrimeScript RT Reagent Kit (Takara Bio, Shiga, Japan). The qRT-PCR was carried out with SYBR® Premix Ex Taq™ II (Perfect Real Time) (Takara Bio, Shiga, Japan), gene specific primer pairs for *PR1*, *PDF1.2* or *ACT2/8*, respectively, and cDNA as template. Arabidopsis *ACT2/8* was used as reference genes. The primers used for qRT-PCR were as previously described (*PR1*[37], *PDF1.2a*[38], and *ACT2/8*[39]). The qRT-PCR analysis was performed using the Mx3000P QPCR System (Agilent Technologies, CA, USA). The following PCR program was used: initial denaturation, 95°C, 10 s; 40 cycles of 95°C for 5 s, 60°C for 20 s and 72°C for 30 s with a temperature transition rate of 20°C/s; and a melting curve analysis, at 95°C for 0 s and 65°C for 15 s, and an increase to 95°C with a temperature transition rate of 0.1°C/s. To generate a standard curve, homologous standards were used in all experiments. The cDNA quantities of target genes were calculated using Mxpro QPCR software (Agilent Technologies, CA, USA). The q-PCR analysis was carried out 3 times.

### In-gel kinase assay

Crude extracts were prepared from *F. sporotrichioides*–inoculated or mock-treated leaves. An in-gel kinase assay was performed as previously described [9].

### Quantification of T-2 toxin

The plant and fungal materials were ground to a fine powder in liquid nitrogen with a mortar and pestle. For T-2 toxin extraction, the fine powder was added to 10 mL of an acetonitrile–water (84:16) solution containing 1 μg/mL diacetoxyscirpenol (DAS) as an internal standard. The solution was then incubated on a rotator for 60 min at room temperature. The extraction mixture was centrifuged (2,000 *g*, at room temperature, for 5 min), and the supernatant collected.

To purify T-2 toxin, we used MycoSep 227 Trich columns (Romer Labs, Inc., MO, USA). The extraction mixture was added to a test tube, and a column was slowly inserted into the test tube. The purified solutions (fraction 1) were collected using the columns and transferred to new tubes. An acetonitrile–water (84:16) solution (3 mL) containing 1 μg/mL DAS was added to the test tube, a column was slowly inserted into the test tube again, and these purified solutions were added to fraction 1. Aliquots of these extraction mixtures were evaporated using a SpeedVac concentrator.

To quantify T-2 toxin, the extracts were mixed with 1 mg/mL sodium azide as a matrix and analyzed using a 4800 Plus MALDI TOF/TOF^TM^ analyzer (AB Sciex, CA, USA).

### Proteomic analysis of Arabidopsis rosette leaves inoculated with *F. sporotrichioides*

The plant materials were ground to a fine powder in liquid nitrogen using a mortar and pestle. For protein extraction, approximately 2 g of this fine powder was added to 5 mL of PBS buffer containing 1% Triton-X 100, 1 mM phenylmethanesulfonyl fluoride (PMSF) and 1/1000 protease inhibitor cocktail (Sigma-Aldrich, MO, USA) and the solution thoroughly mixed by vortexing. The extract was centrifuged (14,000 *g* at 4°C for 15 min) and the supernatant collected. The concentration of protein in each sample was measured with an RC DC Protein Assay Kit (Bio-Rad Japan, Tokyo, Japan). Next, the protein was precipitated with 5% trichloroacetic acid (TCA), and the resulting pellet was washed with 100% acetone at least twice. The acetone was briefly evaporated from the protein by aspiration for 5 min. The protein was subsequently dissolved in lysis buffer (8 M urea, 2% CHAPS, and 30 mM Tris–HCl, pH 8.5). The insoluble material was removed by centrifugation (15,000 *g* at 4°C for 15 min), and the pH of the supernatant (ranging from 8 to 9) confirmed with litmus paper.

The proteins were labeled using CyDye DIGE Fluors developed for fluorescence 2-D technology (GE Healthcare, Tokyo, Japan) according to the manufacturer’s recommendations. Each sample was covalently labeled with a different fluorescent dye: Cy3 (mock) or Cy5 (Arabidopsis inoculated with *F. sporotrichioides*). CyDye-labeled proteins (each 30 μg) were loaded onto an 18-cm rehydration strip with an immobilized pH gradient of 3–10 and separated on a Multiphor electrophoresis unit (GE Healthcare, Tokyo, Japan) using the following setting: a 1.5-h gradient from 300–3,500 V, and 5 h at 3,500 V. After the isoelectric focusing (IEF), the rehydration strip was mounted onto the top of a 10% SDS-polyacrylamide gel with a stacking gel, in a Hoefer SE 600 Ruby system (GE Healthcare, Tokyo, Japan). Electrophoresis was performed at a constant voltage of 10 mA for 7 h. After the 2D electrophoresis, the acrylamide gels were directly scanned using a Typhoon^TM^ 9400 imager (GE Healthcare, Tokyo, Japan). The scanned images were applied to ImageQuant V5.2 software (GE Healthcare, Tokyo, Japan). PDQuest Advanced software (Bio-Rad Japan, Tokyo, Japan) was used to compare individual images and to localize some of the spots. Differentially expressed proteins were identified by Student’s *t*-test of spot intensity (P < 0.05, n = 3). The protein spots were collected from the gels using an Ettan Spot Picker (GE Healthcare, Tokyo, Japan).

### Identification of proteins by MALDI-TOF/TOF analysis

MALDI-TOF/TOF analysis was performed, as previously described [40].

### Functional classification of proteins

The identified proteins were classified according to functional categories using The Plant Proteome Database ( http://ppdb.tc.cornell.edu/default.aspx).

## Competing interests

The authors declare that they have no competing interests.

## Authors’ contributions

TA carried out the experiments. MK isolated *F. graminearum* H3 and ZEA-1 strains. TA and TN designed experiments and wrote the manuscript. TN designed this project. All authors read and approved the final manuscript.

## Supplementary Material

Additional file 1 **Mass spectra of T-2 toxin and diacetoxyscirpenol using MALDI-TOF analysis.** (A) T-2 toxin peaks were detected at m/z 489.2. (B) Peaks of the matrix (sodium azide) only. (C) Diacetoxyscirpenol peaks were detected at m/z 389.1. (D) Peaks of the matrix (sodium azide) only. (TIFF 1721 kb) Click here for file
