# Supplementary figures and images for: The defense response in Arabidopsis thaliana against Fusarium sporotrichioides
Source: Proteome Sci. 2012 Oct 30;10:61. doi: 10.1186/1477-5956-10-61 (PMC3507649; doi:10.1186/1477-5956-10-61)

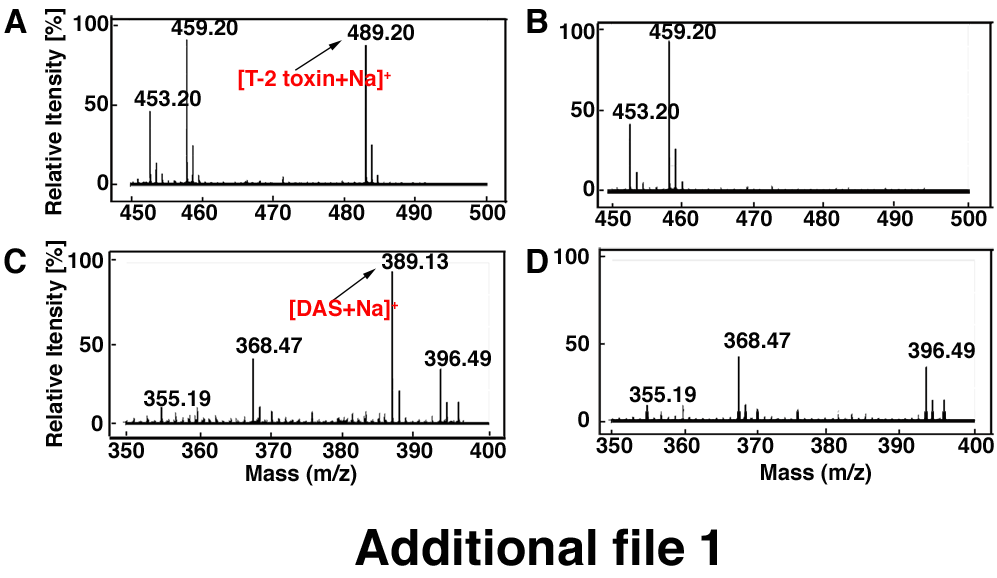

Supplement: Additional file 1 — Mass spectra of T-2 toxin and diacetoxyscirpenol using MALDI-TOF analysis. (A) T-2 toxin peaks were detected at m/z 489.2. (B) Peaks of the matrix (sodium azide) only. (C) Diacetoxyscirpenol peaks were detected at m/z 389.1. (D) Peaks of the matrix (sodium azide) only. (TIFF 1721 kb) [file 1477-5956-10-61-S1.tiff]
